# A Nomogram Based on Preoperative Lab Tests, BMI, ICG-R15, and EHBF for the Prediction of Post-Hepatectomy Liver Failure in Patients with Hepatocellular Carcinoma

**DOI:** 10.3390/jcm12010324

**Published:** 2022-12-31

**Authors:** Deyao Zhang, Yangxun Pan, Zhenyun Yang, Huilan Zeng, Xin Wang, Jinbin Chen, Juncheng Wang, Yaojun Zhang, Zhongguo Zhou, Minshan Chen, Dandan Hu

**Affiliations:** 1 Collaborative Innovation Center for Cancer Medicine, State Key Laboratory of Oncology in South China, Sun Yat-sen University Cancer Center, Guangzhou 510060, China; 2 Department of Liver Surgery, Sun Yat-sen University Cancer Center, Guangzhou 510060, China

**Keywords:** post-hepatectomy liver failure, body mass index, ICG clearance test, effective hepatic blood flow, ICG-R15

## Abstract

**Background:** Liver cancer is one of the most common malignant tumors, and worldwide, its incidence ranks sixth, and its morality third. Post-hepatectomy liver failure (PHLF) is the leading cause of death in patients who have undergone liver resection. This retrospective study investigated the risk factors for PHLF by predicting and constructing an index to evaluate the risk. This was achieved by combining the lab tests with an indocyanine green (ICG) clearance test. **Methods:** The study analyzed 1081 hepatocellular carcinoma (HCC) patients who had received liver resection at Sun Yat-sen University Cancer Center between 2005 and 2020. The patients were divided into a PHLF group (*n* = 113) and a non-PHLF group (*n* = 968), according to the International Study Group of Liver Surgery (ISGLS) criteria. Receiver operating characteristics (ROC) curves were then used to estimate the optimal cut-off values. Univariate and multivariate logistic regression analyses were performed to identify the independent risk factors. Finally, a nomogram was constructed where the calibration plot, the areas under the ROC curve (AUC), and the decision curve analysis (DCA) showed good predictive ability. **Results:** Correlation analysis revealed that body mass index (BMI) was positively correlated with ICG-R15 and with effective hepatic blood flow (EHBF). Univariate and multivariate logistics regression analysis revealed that BMI, ICG-R15, international normalized ratio (INR), tumor size, hepatic inflow occlusion (HIO) time, and operation method were independent predictive factors for PHLF. When these factors and EHBF were included in the nomogram, the nomogram showed a good predictive value, with a C-index of 0.773 (95% Confidence Interval [CI]: 0.729–0.818). The INR had the largest ROC areas (AUC INR = 0.661). Among the variables, ICG-R15 (AUC ICG-R15 = 0.604) and EHBF (AUC EHBF = 0.609) also showed good predictive power. **Conclusions:** The risk of PHLF in HCC patients can be precisely predicted by this model prior to the operation. By integrating EHBF into the model, HCC patients at higher risk for PHLF can be identified more effectively.

## 1. Introduction

Liver cancer is one of the most common malignant tumors, and worldwide, its incidence ranks sixth, and its morality third [1]. Hepatocellular carcinoma (HCC) is the most common pathological subtype of liver cancer, and chronic hepatitis B infection is the leading cause. China has the largest HCC population caused by hepatitis B in the world [2]. Despite recent advances in targeted therapy and immunotherapy for liver cancer, surgical resection remains one of the most important curative treatment strategies.

Previous studies reported that resection of approximately 70% of the total liver volume can be performed safely in patients without liver diseases [3,4]. However, in most liver cancer patients, the cancer is combined with chronic hepatitis viral infection, and severe cirrhosis. This ongoing hepatic structural damage results in a diminished liver functional reserve and an increased risk of post-hepatectomy liver failure (PHLF). Although the imaging measures of residual liver volume, perioperative management, and surgical techniques have been constantly improving, PHLF remains the leading cause of hepatectomy-related mortality [5]. Therefore, it is essential to evaluate residual liver functionality precisely prior to hepatic resection.

Current methods for predicting hepatic functional reserve involve both static and dynamic assessment of liver function. The static liver function assessment mainly involves blood biochemistry and coagulation functions. Research shows that combining different indicators may provide a better reflection of liver function, such as Child-Pugh classification [6], model for end-stage liver disease (MELD) score [7], albumin-bilirubin (ALBI) score [8], and aspartate aminotransferase to platelet ratio index (APRI) [9]. The dynamic liver function assessment includes the indocyanine green (ICG) clearance test [10], the sorbitol clearance test [11], and the lignocaine metabolite (MEGX) liver function test [12]. However, the static liver function assessment has some limitations due to the large reserve of the liver’s functional capacity. In contrast, the dynamic liver function assessment evaluates in real time, quantitatively, and accurately, which is of greater relevance for patients scheduled for liver resection.

The ICG clearance test is the most widely used quantitative liver function test [13,14]. ICG is a tricarbocyanine dye that binds to serum proteins (albumin and lipoproteins). It is taken up by the hepatic parenchymal cells and secreted into the bile in free form. The ICG clearance test comprehensively measures the real-time state of the normal physiological functions of the liver (substance intake, metabolism, synthesis, bioconversion, excretion, and so on) [15]. The ICG clearance test is markedly different from the static biochemical indices used to evaluate the status of impaired liver function. Among the indicators of the ICG test, ICG clearance at 15 min (ICG R15), and the indocyanine green clearance maximum removal rate (ICG Rmax) have been addressed the most, while little attention has been paid to other indicators. Recently, some studies have shown that effective hepatic blood flow (EHBF) is closely related to the severity of acute or chronic liver failure (ACLF) in hepatitis B patients [16]. EHBF, which is the most effective index to evaluate liver ischemia, may be an effective tool for indirectly estimating hepatocellular injury and assessing the liver function reserve. In considering these factors, this study aimed to evaluate the comprehensive indexes of the ICG clearance test as predictors of PHLF and to build a simple, inexpensive, reliable prediction model for use with HCC patients.

## 2. Materials and Methods

### 2.1. Patients

We retrospectively analyzed 1081 HCC patients who have undergone surgical resection at Sun Yat-sen University Cancer Center between April 2005 and April 2020. The inclusion criteria were as follows: (1) received an ICG clearance test 1–3 days before surgery, (2) liver function Child-Pugh A or B, (3) Eastern Cooperative Oncology Group (ECOG) performance status of 0–1, and (4) positive hepatitis B surface antigen. The exclusion criteria included (1) other synchronous malignancies, (2) preoperative obstructive jaundice, (3) a posttreatment survival time of less than one month, (4) incomplete clinical data, and (5) lack of a follow-up assessment.

### 2.2. Definitions

Postoperative PHLF was diagnosed according to the criteria issued by the International Study Group of Liver Surgery (ISGLS) in 2011. It is characterized by an increased international normalized ratio (INR) together with neonatal hyperbilirubinemia (NHB) on or after postoperative day 5 [17]. According to the laboratory-defined normal ranges at our center, bilirubin > 20.5 μmol/L was taken as hyperbilirubinemia, and INR was increased when it was over 1.20.

### 2.3. ICG Clearance Test

The ICG test was carried out noninvasively using a Pulse Dye Densito-Graph Analyzer (DDG-3300K; Nihon-Kohden, Tokyo, Japan). The hemoglobin, height, and weight of patients were measured, and an iodine allergy test was performed before the ICG test. After injecting the ICG (0.5mg/kg; Weichai Pharmaceutical, Shenyang, China) into the peripheral veins of the forearm, a nasal mucosa probe was used to record changes in ICG concentration. Then, the Indocyanine green plasma disappearance rate (ICG-PDR), R15, half-life period (T1/2), and EHBF were assessed by a computer.

### 2.4. Follow-Up and Data

Patients were followed in the first postoperative month, and thereafter, every three months until two years, and then every six months until year five. Patients received radiological examinations such as dynamic computed tomography (CT) scans, magnetic resonance imaging (MRI), and chest radiography on time. Laboratory tests included blood routine, liver function, and liver tumor markers. The overall survival (OS) was calculated from the treatment date to the date of death or to the last follow-up time. 

### 2.5. Statistical Analysis

Statistical analyses were performed using SPSS 19.0 software version 26.0 (IBM Corporation, Armonk, NY, USA) and R version 3.5.1 (R Foundation for Statistical Computing, Vienna, Austria. http://www.r-project.org accessed on 1 September 2021). The Student’s t-test was used to test the normal distribution of continuous variables, and the Mann–Whitney U-test was used to compare the skewed distribution of continuous variables. Pearson’s chi-square test or Fisher’s exact test was used to compare categorical variables. Correlation analysis was performed with Pearson’s correlation test. In addition, receiver operating characteristics (ROC curves) were used to estimate the optimal cut-off values that could be used to divide patients into either the PHLF group or the non-PHLF group. A logistics regression model was then used to analyze the risk factors associated with PHLF, and a nomogram was constructed to predict the occurrence of PHLF. The performance characteristics of the predictive nomogram were evaluated by calibration plots. ROC curves, decision-curve analysis (DCA), and the area under the curve (AUC) were calculated to analyze the predictive accuracy of the nomogram. The test level (alpha) was set at 0.05, and all tests were two-tailed.

## 3. Results

### 3.1. Clinical-Pathological Characteristics

**Figure 1:**
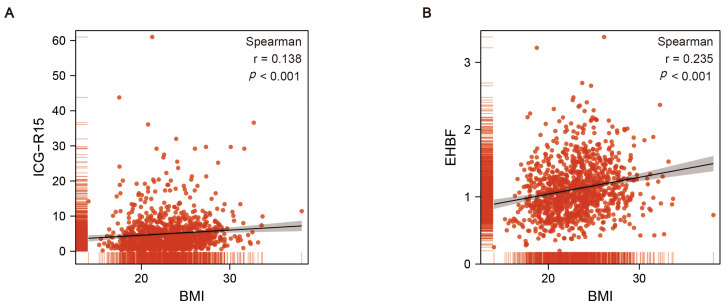
(**A**) Correlation analysis of BMI and ICG-R15. (**B**) Correlation analysis of BMI and EHBF.

A total of 1081 HCC patients who underwent hepatectomy were included in our study, which included 978 males and 103 females, with a median age of 52.2 ± 11.6 years. All patients were HBsAg-positive. The median diameter of the tumor size was 49.2 mm (range, 18.8–79.6 mm). Vascular invasion and extrahepatic metastasis were found in 44 patients (4.1%) and 24 patients (2.2%), respectively. Based on the ISGLS criteria, there were 113 (10.5%) cases of PHLF (PHLF group) and 968 (89.5%) cases without PHLF (non-PHLF group) (Table 1). There were no significant differences in most baseline characteristics between the PHLF group and the non-PHLF group, with the exception of body mass index (BMI), ICG-R15, EHBF, platelet (PLT), albumin (ALB), prothrombin time (PT), activated partial thromboplastin time (APTT), international normalized ratio (INR), operation method, hepatic inflow occlusion (HIO) time, and overall survival time (Table 1). After comparing the differences in the blood biochemical parameters for the two groups, we also performed a correlation analysis between BMI, ICG-R15, and EHBF. Interestingly, the BMI was positively correlated with ICG-R15 and EHBF (*p* < 0.001) (Figure 1).

### 3.2. Cut-Off Value for Clinical Factors

ROC curves were used to estimate the optimal cut-off values for dividing patients into the PHLF group or the non-PHLF group (Supplement Figure S1). Using the Youden index, the optimal cut-off values of age, BMI, ICG-R15, EHBF, PLT, ALB, HGB, ALT, AST, TBIL, DBIL, PT, APTT, INR, tumor size, operation time, blood loss, and HIO time were determined to be 50.5 years, 25.523 kg/m^2^, 2.850%, 0.902 L/min, 167.5 × 10^9^/L, 42.15 mg/dL, 15.05 g/dL, 38.35 U/L, 31.350 U/L, 6.85 μmol/L, 3.55 μmol/L, 12.050 s, 27.250 s, 1.045, 65.5 mm, 88.5 min, 275 mL, and 21.3 min, respectively. Thereafter, the continuous variables were converted into two-class variables.

### 3.3. Univariate and Multivariate Logistic Regression Results

Patients with a BMI > 25.523 showed less risk of PHLF than patients with a BMI ≤ 25.523 (Odds Ratio [OR] = 0.348; 95% Confidence Interval [CI]: 0.179–0.679; *p* = 0.002). Additionally, there was a significant difference between the PHLF group patients and the non-PHLF group patients with respect to ICG-R15 (OR = 2.791; 95% CI: 1.722–4.524; *p* < 0.001) and EHBF in the ICG clearance test (OR = 0.396; 95% CI: 0.267–0.587; *p* < 0.001). Serum blood biochemistry of ALB, alanine aminotransferase (ALT), aspartate aminotransferase (AST), and direct bilirubin (DBIL) was closely related to the occurrence of PHLF (*p* < 0.05). At the same time, preoperative total bilirubin (TBIL) was not significantly correlated with the occurrence of PHLF (*p* > 0.05). Prolonged PT, APTT, and INR were also associated with an increased risk of PHLF (*p* < 0.05). In addition to blood test indicators, the surgical procedure itself could be an explanation for the occurrence of PHLF. Compared with open surgery, the incidence of PHLF was higher following laparoscopy. We also found that shorter HIO time, smaller tumor size, and less blood loss were associated with a lower probability of PHLF (*p* < 0.05), while the anatomical hepatectomy operation did not play a major role in the incidence of PHLF (*p* > 0.05) (Table 2).

**Figure 2:**
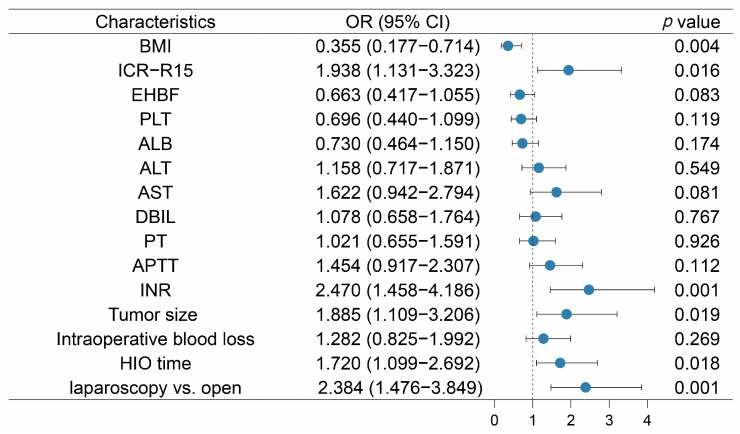
Results of the multivariate logistic regression analysis.

Based on the results from the univariate analysis, BMI, ICG-R15, EHBF, PLT, ALB, ALT, AST, DBIL, PT, APTT, INR, tumor size, intraoperative blood loss, HIO time, and operation method were all included in the multivariate analysis. Regarding coefficients, the operation method was the most significant predictor (OR = 2.384; 95% CI: 1.476–3.849; *p* < 0.001), which was far more significant than any other factor. ICG-R15 (OR = 1.938; 95% CI: 1.131–3.323; *p* = 0.016), INR (OR = 2.470; 95% CI: 1.458–4.186; *p* = 0.001), tumor size (OR = 1.885; 95% CI: 1.109–3.206; *p* = 0.019), and HIO time (OR = 1.720; 95% CI: 1.099–2.692; *p* = 0.018) also had good predictive ability. In addition, EHBF (OR = 0.663; 95% CI: 0.417–1.055; *p* = 0.083) had the potential for predictive utility. Forest plots for each variant are provided in Figure 2.

### 3.4. Nomogram Construction and Validation

**Figure 3:**
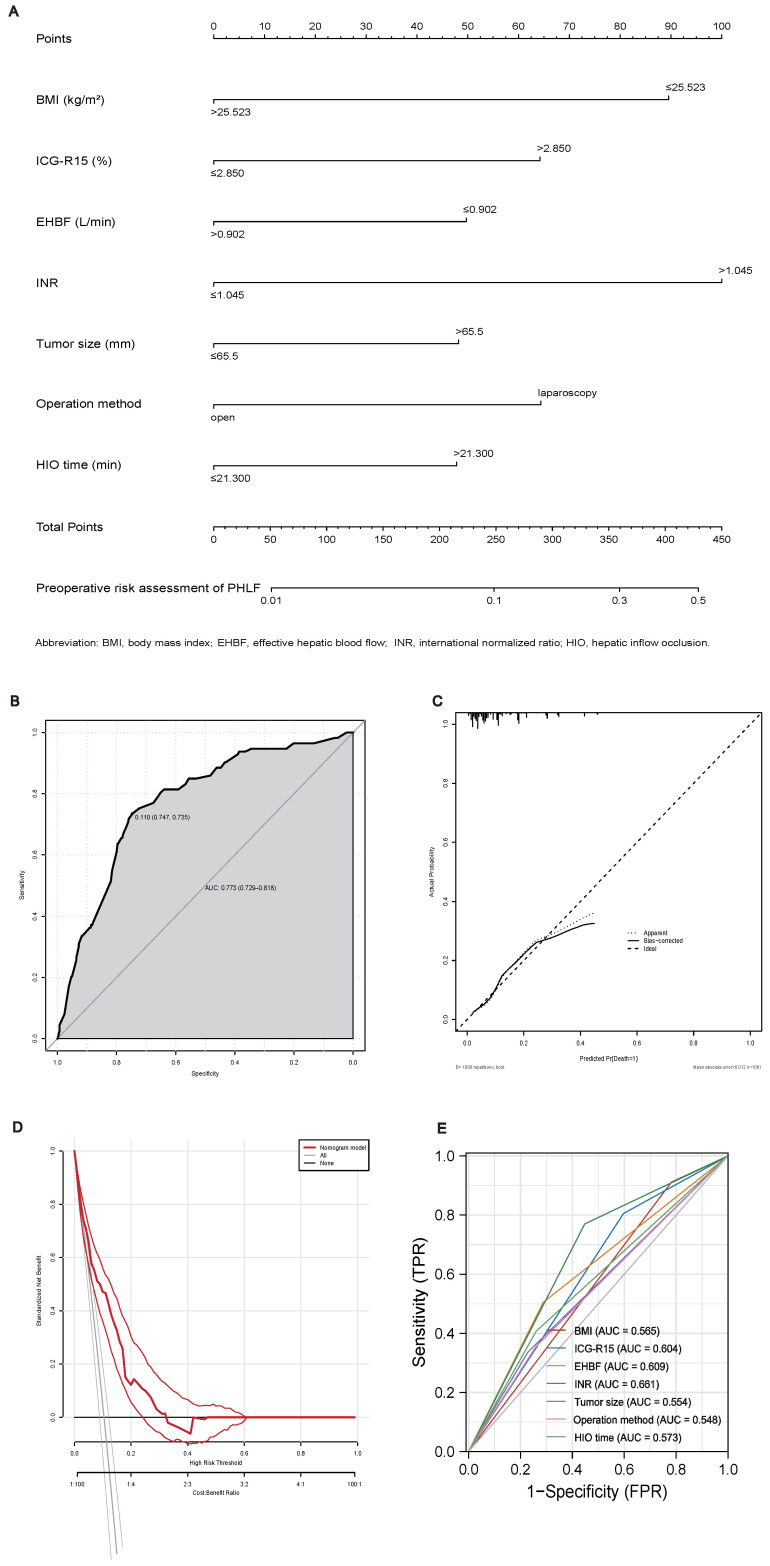
(**A**). Nomogram for preoperative assessment of PHLF. (**B**) Calibration plot of the nomogram. (**C**). ROC curves verified the predictive accuracy of the nomogram. (**D**). Decision-curve analysis (DCA) plot depicting the standardized net benefit. (**E**). The area under the curve (AUC) for BMI, ICG-R15, EHBF, INR, tumor size, operation method, and HIO time.

We constructed a nomogram (Figure 3A) to quantify the results of the logistics regression. INR, the variable with the largest absolute coefficient value, was set as the reference, with a scale range of 0–100 points. With each variable assigned a score, the total score was calculated by summing up these scores of all variables and located it on the total point scale. We could obtain an individualized probability of occurrence of PHLF according to their total points. The C-index of the nomogram was 0.773 (95% CI: 0.729–0.818) (Figure 3B), indicating that the model was well-fitted. The calibration plots for the nomogram were developed internally with bootstrap sampling (*n* = 1000) (Figure 3C), which showed that the nomogram performed well. Finally, we plotted DCA curves to illustrate the discriminating superiority of the nomogram (Figure 3D).

The accuracy of the predicted variables was analyzed using ROC curves (Table 3). ROC analyses showed that a higher risk rating predicted the occurrence rate of PHLF more accurately, with a higher AUC (Figure 3E). The INR exhibited the largest ROC curve area (0.661), and operation method exhibited the smallest ROC curve area (0.548). The AUC of BMI, ICG-R15, and EHBF were 0.565, 0.604, and 0.609, respectively.

## 4. Discussion

Surgical resection continues to be the most effective treatment for early liver cancer patients. Hepatitis B virus (HBV) and hepatitis C virus (HCV) are primary risk factors for chronic hepatitis, liver cirrhosis, and HCC [18]. Allaire et al. reported that, in approximately 86% of HCC patients, cancer was combined with chronic liver disease, especially liver cirrhosis [19]. The presence of liver cirrhosis substantially increases the risks in patients who undergo liver resection, especially for PHLF, which is the leading cause of perioperative mortality and occurs in up to 10% of cases [20]. Treatment of PHLF hinges first on its prevention [21]. Therefore, to improve the safety of surgery and the early recovery of liver function, it is necessary to evaluate the risk of PHLF for each patient.

A full panel of coagulation function must be measured before the procedure, and the potential risk of hemorrhage was thought to be related to the occurrence of PHLF. Previous studies have reported that derangement in INR is associated with poorer prognosis in postoperative patients [22,23], and this has been used for multiple scoring systems, such as the 50-50 criteria [24]. INR was also one of the diagnostic criteria supported by the ISGLS in 2011. In addition, Lei et al. [25] reported that INR was shown to be an independent risk factor for PHLF (OR = 1.07; 95% CI: 1.01–1.12; *p* < 0.05). In our study, INR also had a good predictive ability for PHLF (AUC INR = 0.661).

Additionally, open surgery tended to be associated with a lower risk of PHLF than laparoscopy. This may have been due to the following: The previous study have reported that resection margin width does not predict the survival of HCC patients [26], and that a 1cm margin is usually sufficient [27]. With the help of real-time ultrasound b-scanning, surgeons can perform irregular hepatectomy in order to retain a greater volume of normal liver tissue. When performing a laparoscopic hepatectomy, surgeons tend to choose regular hepatectomy (left hepatectomy, right hepatectomy, and mesohepatectomy), which may result in greater loss in the volume of normal liver tissue. Preserving a greater volume of normal liver tissue may, to some extent, reduce the risk of PHLF.

The ICG clearance test is the most widely used quantitative liver function test in eastern countries [28,29]. The existing studies have mainly focused on the ICG-R15. Sunagawa et al. concluded that ICG-R15 was an independent predictive factor for PHLF (relative risk [RR] = 26.04, *p* = 0.012 < 0.05) [30]. Poon et al. also reported that extended left or right hepatectomy could be safe in patients with Child-Pugh class A with an ICG-R15 of up to 20% [31]. Wang et al. obtained similar results that ICG-R15 predicted PHLF more accurately than Child-Pugh and MELD scores [32]. In our study, ICG-R15 (OR = 1.938; 95% CI: 1.131–3.323; *p* = 0.016) was shown to have a nice predictive ability for PHLF, and ICG-R15 yielded a ROC-plot AUC value of 0.604. In addition, a higher BMI tended to be a protective factor against PHLF (OR = 0.355; 95% CI: 0.177–0.714; *p* = 0.004). This result agrees with that of a previous study by Fahira et al. [33], who reported that the HCC-related mortality rate was lower in patients with a higher BMI than in patients with a lower BMI (Hazard Ratio [HR] = 0.347; 95% CI: 0.239–0.302; *p* < 0.05). 

To the best of our knowledge, this report is the first to show that EHBF could be a significant variable in the preoperative risk assessment for PHLF. In this study, univariate analysis showed that, on average, patients in the non-PHLF group had greater EHBF index values than patients in the PHLF group. Unfortunately, the multivariate analysis showed only a potential trend (OR = 0.663; 95% CI: 0.417–1.055; *p* = 0.083). Taking the correlation between EHBF and BMI into account (Figure 1), we included EHBF in our nomogram analysis. The predictive scores for EHBF were 50 points. In addition, the EHBF obtained better AUC values than R15 or BMI (AUC EHBF = 0.609, AUC R15 = 0.604, AUC BMI = 0.565). However, this difference was not statistically significant (*p*  =  0.083), which may be explained by the following: It needs to be pointed out that the value of EHBF is calculated from the value of blood volume (BV) plus the value of K (the attenuation coefficient of ICG concentration). For patients with a high BMI, BMI does not provide accurate information about body fat distribution [34]. Importantly, the distribution of fat could affect the current density distribution [35], which could result in the inaccurate measurement of BV and EHBF. This also explains the reduced predictive value of EHBF for PHLF. By considering the correlation among BMI, ICG-R15, and EHBF, a comprehensive nomogram could improve the predictive accuracy.

The nomogram was developed using HCC patients whose liver function recovery status was already known. A safer treatment method could be adopted for some new patients whose preoperative examination indicated a high risk of PHLF. Surgeons could improve a patient’s liver function or take effective means for shrinking tumors prior to an operation. During an operation, it is crucial to reduce intraoperative bleeding, HIO time, and surgical trauma, all with the intention of ensuring a therapeutic effect. However, we acknowledge there are some limitations in our study. This is a single-center retrospective study that investigated potential predictors for PHLF. With the development of individualized treatment and enhanced recovery after surgery (ERAS) protocols, the diagnosis and treatment method for PHLF may now be different, so the prediction model may be biased. In addition, all patients in this study had chronic HBV infection, which is different from the major contributors in western countries. This could limit this nomogram’s widespread application. In the future, we plan to enlarge our cohort and design a multicenter study to create a more accurate clinical prediction model for PHLF. 

## 5. Conclusions

BMI, INR, ICG-R15, and surgical procedure together form an effective predictor for PHLF in HCC patients undergoing liver resection. Integrating EHBF into the model could improve the identification of patients at higher risk.

## Figures and Tables

**Table 1:** Baseline clinicopathological characteristics of the patients.

Patient Characteristics	Total (*n* = 1081)	Non-PHLF Group (*n* = 968)	PHLF Group (*n* = 113)	*p*-Value
Age (years)	52.2 ± 11.6	52.1 ± 11.7	53.2 ± 11.4	0.341
Gender	978/103	880/88	98/15	0.206
(male/female, %)	(90.5/9.5)	(90.9/9.1)	(86.7/13.3)	
BMI (kg/m^2^)	23.0 ± 3.2	23.1 ± 3.2	22.4 ± 3.0	0.028
ICG-R15 (%)	5.0 ± 5.1	4.7 ± 4.6	7.3 ± 7.8	<0.001
EHBF (L/min)	1.1 ± 0.4	1.1 ± 0.4	1.0 ± 0.4	<0.001
Child-Pugh classifications	1056 (97.7)	945 (97.6)	111 (98.2)	0.940
(A/B, %)	/25 (2.3)	/23 (2.4)	/2 (1.8)	
PLT (×10^9^/L)	190.7 ± 71.1	192.5 ± 70.3	174.7 ± 76.2	0.011
ALB (mg/dL)	43.1 ± 3.9	43.2 ± 3.7	41.6 ± 4.4	<0.001
HGB (g/dL)	14.9 ± 4.1	14.9 ± 4.3	14.5 ± 1.9	0.319
ALT (U/L)	42.7 ± 36.5	42.6 ± 37.8	43.5 ± 22.7	0.799
AST (U/L)	39.2 ± 29.2	38.8 ± 29.7	43.0 ± 23.6	0.143
TBIL (μmol/L)	13.9 ± 5.7	13.9 ± 5.8	13.6 ± 5.2	0.565
DBIL (μmol/L)	4.6 ± 2.1	4.6 ± 2.1	4.8 ± 2.0	0.283
PT (s)	12.1 ± 1.3	12.0 ± 1.3	12.6 ± 1.0	<0.001
APTT (s)	27.6 ± 4.2	27.5 ± 4.1	28.9 ± 4.4	0.001
INR	1.1 ± 0.1	1.1 ± 0.1	1.1 ± 0.1	<0.001
Tumor size (mm)	49.2 ± 30.4	48.6 ± 30.1	53.6 ± 33.1	0.102
Tumor numbers	855 (79.1)	773 (79.9)	82 (72.6)	0.093
(single/multiple, %)	/226 (20.9)	/195 (20.1)	/31 (27.4)	
Presence vascular invasion	44 (4.1)	41 (4.2)	3 (2.7)	0.580
(±, %)	/1037 (95.9)	/927 (95.8)	/110 (97.3)	
Presence extrahepatic metastasis (±, %)	24 (2.2%)	22 (2.3)	2 (1.8)	0.995
	/1057 (97.8)	/946 (97.7)	/111 (98.2)	
BCLC staging (A/B/C, %)	856 (79.2)	765 (79.0)	91 (80.5)	0.932
	/144 (13.3)	/130 (13.4)	/14 (12.4)	
	/81 (7.5)	/73 (7.5)	/8 (7.1)	
Presence of ascites	16 (1.5)	13 (1.3)	3 (2.7)	0.496
(±, %)	/1065 (98.5)	/955 (98.7)	/110 (97.3)	
MVI	330 (30.5)	287 (29.6)	43 (38.1)	0.066
(±, %)	/751 (69.5)	/681 (70.4)	/70 (61.9)	
Operation time (min)	159.3 ± 57.1	159.6 ± 57.2	156.0 ± 56.0	0.525
Operation method	290 (26.8)	250 (25.8)	40 (35.4)	0.039
(laparoscopy/open, %)	/791 (73.2)	/718 (74.2)	/73 (64.6)	
Intraoperative blood loss (mL)	321.7 ± 386.5	315.4 ± 379.6	375.4 ± 438.9	0.118
HIO time (min)	13.7 ± 13.2	13.4 ± 13.1	16.0 ± 13.6	0.047
Anatomical hepatectomy (yes/no, %)	131 (12.1)	119 (12.3)	12 (10.6)	0.606
	/950 (87.9)	/849 (87.7)	/101 (89.4)	
Overall survival (months)	30.0 ± 31.6	31.5 ± 32.6	17.6 ± 17.1	<0.001

Abbreviation: BMI, body mass index; EHBF, effective hepatic blood flow; PLT, platelet; ALB, albumin; HGB, hemoglobin; ALT, alanine aminotransferase; AST, aspartate aminotransferase; TBIL, total bilirubin; DBIL, direct bilirubin; PT, prothrombin time; APTT, activated partial thromboplastin time; INR, international normalized ratio; BCLC, Barcelona clinic liver cancer; MVI, microvascular invasion; HIO, hepatic inflow occlusion; PHLF, post-hepatectomy liver failure.

**Table 2:** Univariate and Multivariate analysis for PHLF.

	Univariate Analysis		Multivariate Analysis	
	Odds Ratio (95% CI)	*p*-Value	Odds Ratio (95% CI)	*p*-Value
Age (years)	0.875	0.503		
>50.500 vs. ≤50.500	(0.592–1.293)			
Gender	1.531	0.154		
Male vs. female	(0.852–2.750)			
BMI (kg/m^2^)	0.348	0.002	0.355	0.004
>25.523 vs. ≤25.523	(0.179–0.679)		(0.177–0.714)	
ICG-R15 (%)	2.791	<0.001	1.938	0.016
>2.850 vs. ≤2.850	(1.722–4.524)		(1.131–3.323)	
EHBF (L/min) >0.902 vs. ≤0.902	0.396 (0.267–0.587)	<0.001	0.663	0.083
			(0.417–1.055)	
Child-Pugh classifications	1.351	0.686		
B vs. A	(0.314–5.806)			
PLT(×10^9^/L) >167.500 vs. ≤167.500	0.516 (0.348–0.764)	0.001	0.696	0.119
			(0.440–1.099)	
ALB (mg/dL) >42.150 vs. ≤42.150	0.460 (0.311–0.682)	<0.001	0.730	0.174
			(0.464–1.150)	
HGB (g/L)	0.688	0.071		
>150.050 vs. ≤150.050	(0.459–1.033)			
ALT (U/L)	1.784	0.004	1.158	0.549
>38.350 vs. ≤38.350	(1.206–2.640)		(0.717–1.871)	
AST (U/L)	2.919	<0.001	1.622	0.081
>31.350 vs. ≤31.350	(1.887–4.515)		(0.942–2.794)	
TBIL (μmol/L)	2.206	0.280		
>6.850 vs. ≤6.850	(0.524–9.276)			
DBIL (μmol/L)	1.610	0.039	1.078	0.767
>3.550 vs. ≤3.550	(1.024–2.531)		(0.658–1.764)	
PT (s)	1.635	0.014	1.021	0.926
>12.050 vs. ≤12.050	(1.106–2.418)		(0.655–1.591)	
APTT (s)	2.225	<0.001	1.454	0.112
>27.250 vs. ≤27.250	(1.477–3.354)		(0.917–2.307)	
INR	4.134	<0.001	2.470	0.001
>1.045 vs. ≤1.045	(2.620–6.523)		(1.458–4.186)	
Tumor size (mm)	1.713	0.012	1.885	0.019
>65.5 vs. ≤65.5	(1.127–2.602)		(1.109–3.206)	
Tumor numbers	1.499	0.073		
Multiple vs. single	(0.963–2.332)			
Presence vascular invasion	0.617	0.425		
Yes vs. no	(0.188–2.024)			
Presence extrahepatic Metastasis Yes vs. no	0.775 (0.180–3.339)	0.732		
BCLC staging B vs. A C vs. A	Ref. 0.905 (0.501–1.637) 0.921 (0.430–1.973)	0.742 0.833		
Presence of ascites	2.003	0.284		
Yes vs. no	(0.562–7.140)			
MVI	1.458	0.068		
Yes vs. no	(0.973–2.183)			
Operation time (min)	1.955	0.266		
>88.500 vs. ≤88.500	(0.599–6.377)			
Intraoperative blood loss (mL)	1.735	0.006	1.282	0.269
>275.000 vs. ≤275.000	(1.174–2.566)		(0.825–1.992)	
HIO time (min)	1.951	0.001	1.720	0.018
>21.300 vs. ≤21.300	(1.305–2.915)		(1.099–2.692)	
Anatomical hepatectomy	0.848	0.606		
Yes vs. no	(0.452–1.589)			
Operation method	1.574	0.031	2.384	<0.001
Laparoscopy vs. open	(1.043–2.375)		(1.476–3.849)	

Abbreviation: BMI, body mass index; EHBF, effective hepatic blood flow; PLT, platelet; ALB, albumin; HGB, hemoglobin; ALT, alanine aminotransferase; AST, aspartate aminotransferase; TBIL, total bilirubin; DBIL, direct bilirubin; PT, prothrombin time; APTT, Activated partial thromboplastin time; INR, international normalized ratio; BCLC, Barcelona clinic liver cancer; MVI, microvascular invasion HIO, hepatic inflow occlusion.

**Table 3:** The accuracy of predicted variables.

Variable	AUC (95%)	Sensitivity	Specificity	Positive Predictive Value	Negative Predictive Value	Youden Index
BMI	0.565 0.535–0.594	0.912	0.218	0.120	0.955	0.129
ICG-R15	0.604 0.564–0.644	0.805	0.403	0.136	0.947	0.208
EHBF	0.609 0.560–0.657	0.504	0.713	0.170	0.925	0.217
INR	0.661 0.619–0.703	0.770	0.553	0.167	0.954	0.323
Tumor size	0.554 0.508–0.594	0.336	0.772	0.147	0.909	0.108
Operation method	0.548 0.501–0.594	0.354	0.742	0.138	0.908	0.096
HIO time	0.573 0.526–0.621	0.407	0.740	0.154	0.914	0.147

Abbreviation: BMI, body mass index; EHBF, effective hepatic blood flow; INR, international normalized ratio; HIO, hepatic inflow occlusion.

## Data Availability

Not applicable; anonymized data will be supplied upon request to the corresponding author.

## Supplementary Materials

The following supporting information can be downloaded at: https://www.mdpi.com/article/10.3390/jcm12010324/s1, Supplement Figure S1: Cut-off identification by ROC curve of (A) Age, (B) BMI, (C) ICG-R15, (D) EHBF, (E) PLT, (F) ALB, (G) HGB, (H) ALT, (I) AST, (J) TBIL, (K) DBIL, (L) PT, (M) APTT, (N) INR, (O) Tumor size, (P) Operation time, (Q) Intraoperative blood loss, (R) HIO time.
