# Supplementary material for: A Nomogram Based on Preoperative Lab Tests, BMI, ICG-R15, and EHBF for the Prediction of Post-Hepatectomy Liver Failure in Patients with Hepatocellular Carcinoma
Source: J Clin Med. 2022 Dec 31;12(1):324. doi: 10.3390/jcm12010324 (PMC9821206; doi:10.3390/jcm12010324)
Supplement: Supplementary file 1 [file jcm-12-00324-s001.zip › jcm-2041892-supplementary.pdf]

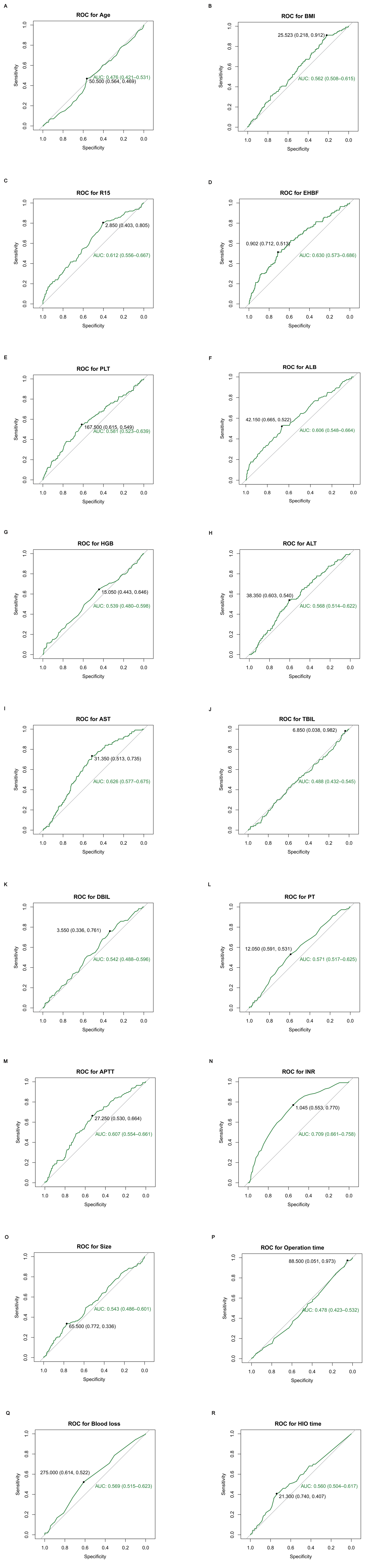

Supplement Figure S1: Cut-off identification by ROC curve of (A) Age, (B) BMI, (C) ICG-R15, (D) EHBF, (E) PLT, (F) ALB, (G) HGB, (H) ALT, (I) AST, (J) TBIL, (K) DBIL, (L) PT, (M) APTT, (N) INR, (O) Tumor size, (P) Operation time, (Q) Intraoperative blood loss, (R) HIO time.
